# A Comparison of South African National HIV Incidence Estimates: A Critical Appraisal of Different Methods

**DOI:** 10.1371/journal.pone.0133255

**Published:** 2015-07-31

**Authors:** Thomas Rehle, Leigh Johnson, Timothy Hallett, Mary Mahy, Andrea Kim, Helen Odido, Dorina Onoya, Sean Jooste, Olive Shisana, Adrian Puren, Bharat Parekh, John Stover

**Affiliations:** 1 Human Sciences Research Council, Cape Town, South Africa; 2 Centre for Infectious Disease Epidemiology and Research, School of Public Health and Family Medicine, University of Cape Town, Cape Town, South Africa; 3 Department of Infectious Disease Epidemiology, Imperial College London, London, United Kingdom; 4 UNAIDS, Geneva, Switzerland; 5 Centers for Disease Control and Prevention, Center for Global Health, Division of Global HIV/AIDS, Atlanta, GA, United States of America; 6 UNAIDS, Pretoria, South Africa; 7 Department of Psychiatry and Mental Health, University of Cape Town, Cape Town, South Africa; 8 National Institute for Communicable Diseases, Johannesburg, South Africa; 9 Futures Institute, Glastonbury, CT, United States of America; National Center for AIDS/STD Control and Prevention, China CDC, CHINA

## Abstract

**Background:**

The interpretation of HIV prevalence trends is increasingly difficult as antiretroviral treatment programs expand. Reliable HIV incidence estimates are critical to monitoring transmission trends and guiding an effective national response to the epidemic.

**Methods and Findings:**

We used a range of methods to estimate HIV incidence in South Africa: (i) an incidence testing algorithm applying the Limiting-Antigen Avidity Assay (LAg-Avidity EIA) in combination with antiretroviral drug and HIV viral load testing; (ii) a modelling technique based on the synthetic cohort principle; and (iii) two dynamic mathematical models, the EPP/Spectrum model package and the Thembisa model. Overall, the different incidence estimation methods were in broad agreement on HIV incidence estimates among persons aged 15-49 years in 2012. The assay-based method produced slightly higher estimates of incidence, 1.72% (95% CI 1.38 – 2.06), compared with the mathematical models, 1.47% (95% CI 1.23 – 1.72) in Thembisa and 1.52% (95% CI 1.43 – 1.62) in EPP/Spectrum, and slightly lower estimates of incidence compared to the synthetic cohort, 1.9% (95% CI 0.8 – 3.1) over the period from 2008 to 2012. Among youth aged 15-24 years, a declining trend in HIV incidence was estimated by all three mathematical estimation methods.

**Conclusions:**

The multi-method comparison showed similar levels and trends in HIV incidence and validated the estimates provided by the assay-based incidence testing algorithm. Our results confirm that South Africa is the country with the largest number of new HIV infections in the world, with about 1 000 new infections occurring each day among adults aged 15-49 years in 2012.

## Introduction

Incidence estimates provide critical insights into the dynamics of the HIV epidemic and are the most direct means of assessing the impact of HIV prevention programmes. The wide-scale implementation of prevention and treatment interventions means that, more than ever, real-time estimates of HIV incidence levels and trends are essential for evaluating the epidemic trajectory, to inform a more efficient and effective response to both the national and global HIV pandemic [1].

Southern Africa remains the region most severely affected by the HIV epidemic. With over six million people living with HIV/AIDS, South Africa has the largest population of HIV- infected individuals in the world, representing a quarter of the estimated HIV infections in sub-Saharan Africa [1]. It is therefore fitting that South Africa has implemented comprehensive national HIV surveillance efforts with the annual antenatal surveys and repeated national HIV household surveys as the core components for monitoring the HIV epidemic. Antenatal surveillance in South Africa has been carried out since 1990 [2] and there have been four large nationally representative household-based surveys, in 2002, 2005, 2008 and 2012 [3].

As the epidemic matured in South Africa and as access to antiretroviral treatment (ART) was rapidly scaled up in the period post-2004, the interpretation of HIV prevalence trends became increasingly complex. ART has increased the survival time of people living with HIV, with the result that HIV prevalence has increased. Hence, measuring ART coverage and estimating HIV incidence at the population level are critical to assessing the impact of treatment and prevention programs on HIV prevalence. Population-based survey methodology has advanced to address these evolving data needs in South Africa. The inclusion of novel laboratory methodologies in the survey protocol has enabled direct estimation of exposure to ART among HIV-positive individuals as well as direct assay-based HIV incidence measures from cross-sectional blood specimens [4]. The prevalence data from the repeated national HIV household surveys were also ideally suited to estimate HIV incidence using a mathematical approach [5].

Incidence assays, which can discriminate those who have been infected recently from others previously infected with HIV, can indicate recent changes in HIV incidence at a population level. Direct assay-based incidence measures using blood samples also provide incidence estimates by risk categories and for selected sub-populations. However, incidence estimation based on testing cross-sectional blood specimens is often established on relatively small numbers of persons that are classified as recently infected, which means that estimates so derived have large confidence intervals. Furthermore, incidence assays should be used in an algorithm where assay-recent specimens are further tested for additional markers such as ART exposure and HIV viral load, to exclude ‘false recent’ results [6, 7].

Mathematical models, such as the Estimates and Projection Package (EPP) / Spectrum [8, 9] and Thembisa [10], can also generate estimates of incidence by leveraging household survey data together with data from antenatal clinic surveillance and information on survival patterns of people living with HIV (and behaviours, in the case of Thembisa). However, there is a risk, in these models, that biases in the estimates can be introduced when certain assumptions do not hold, and as the method is based on using prevalence data, sudden changes in incidence will not be detected rapidly, since such changes only manifest in prevalence levels after a considerable delay. The incidence trajectory in the Spectrum model is fairly flexible, but irregular patterns in the scale-up of ART can induce severe deviations in the estimated incidence trend. Meanwhile, the incidence trajectory in Thembisa is constrained to follow a path dictated by the patterns of risk assumed, for which some reliance is placed upon a simplified scheme of sexual behaviour derived from self-reported data, which may be inaccurate [11]. Finally, simpler modelling techniques based on the synthetic cohort principle, which compare only age-specific prevalence levels at two points in time, can also be used to generate estimates of incidence [12, 13]. However, such methods also have to rely on many assumptions, especially in the era of ART, which leads to highly uncertain estimates and biases where relative levels of incidence across age-groups are not stable.

As each of these methods has different strengths and weaknesses, a synthesis of the different approaches can generate useful insights into the estimates of HIV incidence in a given setting, as well as revealing information about the performance of the methods. In this paper we compare the different methods to estimate HIV incidence in South Africa in order to arrive at an agreement on estimates of incidence. We also compared the performance of the different components of the assay-based incidence testing algorithm to assess the effect of antiretroviral drug testing and viral load testing on direct incidence measures in cross-sectional blood samples.

## Methods

### Ethics Statement

The survey protocols were approved by the Human Sciences Research Council’s Ethics Committee. All information collected from study participants was anonymized and de-identified prior to analysis. Written informed consent was obtained from study participants and only samples from individuals who consented to have their samples used for future research were used in the cross-sectional incidence testing investigation. The research was conducted according to the principles expressed in the Declaration of Helsinki.

### Survey data

Our incidence estimation methods used nationally representative data collected in HIV household surveys conducted in South Africa in 2005, 2008, and 2012. The surveys applied a multi-stage stratified sampling design with data weighting procedures taking into account the complex sampling design and adjusting for HIV testing non-response. The surveys collected data not only on HIV status but also information on socio-demographic and behavioural characteristics of the South African population, for each sex, age group, race, locality type and province. The 2012 survey included testing for antiretroviral drugs and HIV incidence, providing direct estimates of age- and sex-specific ART exposure and new HIV infections in the South African population [3].

### Direct HIV incidence estimates: HIV incidence testing algorithm

**Fig 1:**
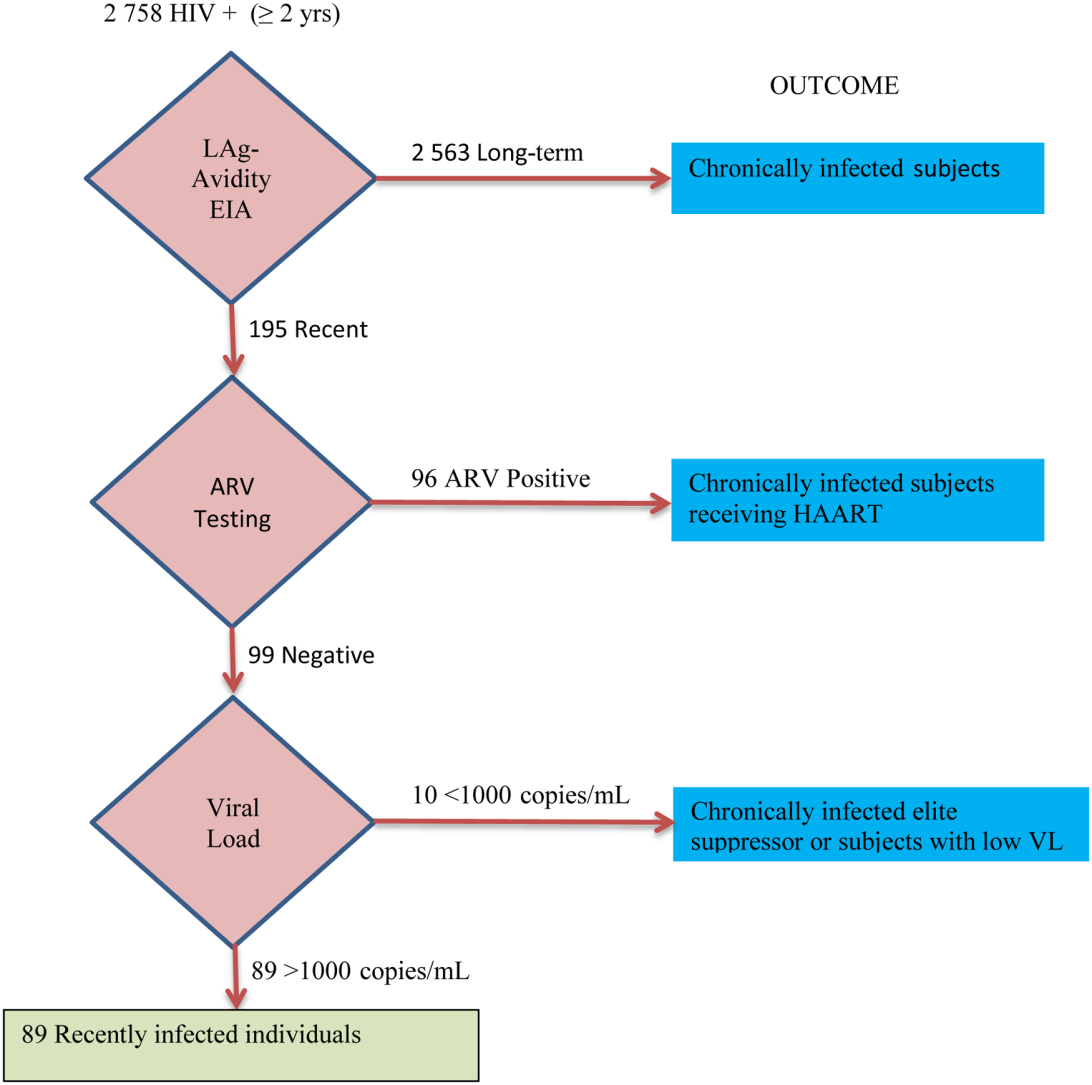
Testing algorithm for recent infection. The figure shows the multi-assay algorithm that was applied for the 2012 HIV incidence estimation on confirmed HIV-positive samples from individuals 2 years and older. The algorithm used the Limiting-Antigen Avidity assay (LAg-Avidity EIA) in combination with testing for antiretroviral drugs (ARV) and HIV-1 viral load (VL copies/mL).

The detection of recent infections was performed on confirmed HIV-positive samples from survey respondents aged 2 years and older. Fig 1 shows the recent infection testing algorithm we applied for the 2012 HIV incidence estimation. The HIV incidence testing algorithm used the Limiting-Antigen Avidity Assay (LAg-Avidity EIA, Maxim Biomedical Inc., Rockville, MD, USA) with a cutoff normalized optical density (ODn) of 1.5 [14, 15] in combination with additional information on antiretroviral treatment exposure and HIV-1 RNA viral load (Abbott m2000 HIV Real-Time System, Abbott Molecular Inc, Des Plaines, IL, USA). The presence of the antiretroviral drugs Zidovudine, Nevirapine, Efavirenz, Lopinavir, Atazanavir and Darunavir was confirmed by means of High Performance Liquid Chromatography coupled to Tandem Mass Spectrometry; the limit of detection was set to 0.2 μg/ml for each of the drugs.

2 758 HIV-positive samples were subjected to HIV incidence testing (Fig 1), 195 specimens were identified as LAg-Avidity EIA recent and 2 563 specimens were determined as non-recent infections. LAg-Avidity EIA recent specimens which tested positive for antiretroviral drugs (n = 96) were considered chronically infected individuals on treatment. Ten out of 99 LAg-Avidity EIA recent, ARV-negative specimens had an HIV viral load < 1 000 copies/ mL (LAg +/ARV-/VL<1 000) and were classified as long-term infections found in elite suppressors or in individuals maintaining a low viral load. Only 89 LAg-Avidity EIA recent, ARV-negative specimens with an HIV viral load > 1 000 copies/ mL (LAg +/ARV-/VL>1 000) were classified as recently-infected individuals in this multi-assay algorithm.

HIV incidence calculations were performed as proposed by the WHO Technical Working Group on HIV Incidence Assays [6, 16]. Incidence was calculated as an annual instantaneous rate. HIV incidence estimates were based on weighted samples to take into account the survey design and adjusted to account for specimens with missing LAg-Avidity EIA test results. Confidence intervals were computed applying a design effect (DEFT) of 2.0 [3]. The mean duration of recent infection (MDRI) was specified as 130 days in the incidence formula [15]. No adjustment factor for false recent results was applied to the incidence calculation based on this multi-assay testing algorithm.

### Mathematically derived incidence from sequential household surveys

An existing method to estimate the average annual HIV-incidence rate in the interval between two surveys was used [12, 13]. A correction was applied that accounted for the effect of ART on HIV prevalence due to increased survival of HIV-infected persons [13, 5]. This required assumptions about the scale-up of ART [17, 18], mortality rates on ART [19] and the mean survival time without ART from the point of ART initiation [20]. A sigmoid time-trend was assumed for the latter, with mean survival increasing from between 0.8 and 1.8 years in 2005 to between 2.0 and 5.1 years in 2012. Bootstrapping was used to reflect sampling uncertainty in the prevalence measurements and parametric uncertainty in the ART correction procedure [21]. Point estimates were the means of the generated distributions and the intervals span the 2.5th to the 97.5th percentiles. Updates in assumptions about the impact of ART on survival and a fuller representation of uncertainties in the present method meant that estimates for the period 2005–2008 were slightly modified from earlier presentations [5].

### EPP/Spectrum model

Incidence was estimated in Spectrum through the EPP model developed by East/West Center [8]. Briefly, HIV prevalence data from antenatal clinic surveillance was used to determine the trends in prevalence over time using a Bayesian melding statistical model [22, 23]. However since pregnant women attending antenatal clinics are not representative of the adult population, the level of the prevalence curve was determined by the household surveys. The household surveys also informed the shape of the prevalence curve. The model estimated incidence trajectories that were consistent with the prevalence data taking into consideration survival of people living with HIV (including whether or not they are receiving ART).

### Thembisa model

Thembisa is a model of the South African HIV epidemic, described elsewhere [10]. Briefly, the model stratified the population by demographic characteristics (age and sex), sexual behaviour characteristics (marital status, risk group and sexual experience), engagement in HIV prevention programmes (history of HIV testing and male circumcision status) and HIV disease stage (HIV-positive individuals were stratified by CD4 count if untreated, and by baseline CD4 count and ART duration if treated). Assumptions regarding sexual behaviour and changes in behaviour over time were based on reviews of South African sexual behaviour data [24, 25], and assumptions regarding changes over time in HIV testing and ART uptake were based on reported rates of HIV testing [3] and reported numbers of ART patients [18]. The model was fitted to age-specific HIV prevalence data from antenatal surveys and household surveys, as well as age-specific reported death data, using a Bayesian procedure. Parameters varied in the model fitting procedure included rates of partnership formation, probabilities of HIV transmission per act of sex, rates of HIV-related mortality and CD4 decline, and the percentage reduction in unprotected sex following an HIV-positive diagnosis.

A comparison of key model inputs and assumptions used by EPP/Spectrum and Thembisa is provided in Table 1.

**Table 1:** Comparison of EPP/Spectrum and Thembisa: Model inputs and assumptions for adult HIV incidence estimation.

	EPP/Spectrum	Thembisa
Data sources used in calibration	HIV prevalence data from ANCs from 1990–2012 are included in the model to develop a prevalence curve over time. Results from the 2005, 2008, 2012 HSRC surveys are included to calibrate the ANC results and inform trends.	Age-specific HIV prevalence data from 1991–2011 ANC surveys, age- and sex-specific prevalence data from 2005, 2008 and 2012 HSRC surveys, self-reported HIV testing data from the same three HSRC surveys, reported death data by age and sex (1997–2010).
Uncertainty analysis	Uncertainty in EPP is quantified using a Bayesian approach, with prior distributions for each of the EPP parameters, a likelihood function based on the listed data sources, and a posterior distribution simulated using IMIS. Uncertainty in Spectrum is calculated by 1000 runs, whereby each run randomly selects an item from the posterior incidence estimate and a set of parameters from the possible ranges of those parameters.	Uncertainty is quantified using a Bayesian approach. Prior distributions are specified to represent ranges of uncertainty around the sexual behaviour, HIV survival, HIV transmission and HCT parameters. A likelihood function is specified based on the data sources listed. The posterior distribution is simulated using IMIS.
Sexual behaviour	Sexual behaviour modelled in EPP by dividing sexually active population into high risk and “not at risk” populations. HIV incidence estimates from EPP are entered into Spectrum, so there are no sexual behaviour assumptions in Spectrum.	Model includes assumptions about % in high risk group, sexual debut, non-marital sex, marriage, divorce, widowhood, commercial sex, mixing between age groups and risk groups, coital frequency, effect of HIV status knowledge and ART on sexual behaviour.
HIV transmission	ART reduces probability of transmission by 70%.	Transmission probability depends on HIV disease stage, type of relationship, age and risk groups of both partners (implicit allowance for STI cofactors). ART reduces HIV transmission probability by 80%.
Untreated HIV survival	HIV-infected adults progress through a 7-stage model of CD4 decline, with HIV-related mortality occurring in all HIV states. Rates of CD4 decline increase with age.	After an initial acute phase, HIV-infected adults progress through a 4-stage model of CD4 decline, with HIV-related mortality occurring in the final two HIV states (CD4 200–349 and CD4 <200). Rates of CD4 decline increase with age.
Condom usage	Not modelled, though there is implicit allowance in EPP for changes in sexual risk behaviour over time.	Probability of condom use depends on age, sex and relationship type. Condom usage in HIV-negative individuals increases over time but reduces after 2008. Condom usage in HIV-positive individuals increases after diagnosis.
Male circumcision	Not modelled.	Model allows for traditional as well as medical male circumcision. Probability of female-to-male transmission is reduced by 60% if male is circumcised.
ART	Coverage estimates based on estimates of total numbers receiving ART in each year. Eligibility criteria change over time, with CD4 350 criterion being applied from mid-2011. Survival assumptions are based on data from IeDEA collaboration (mortality specified separately for 0–6 months, 6–12 months and >12 months after ART initiation).	Coverage estimates based on published estimates of total numbers starting ART in each year, combining public sector data and private sector data. Eligibility criteria change over time, with CD4 350 criterion being applied from mid-2011. Survival assumptions are based on models fitted to data from IeDEA Southern Africa (mortality specified separately for each of first five years of ART).

Abbreviations: EPP: Estimates and Projection Package; ART: antiretroviral therapy.

## Results

**Fig 2:**
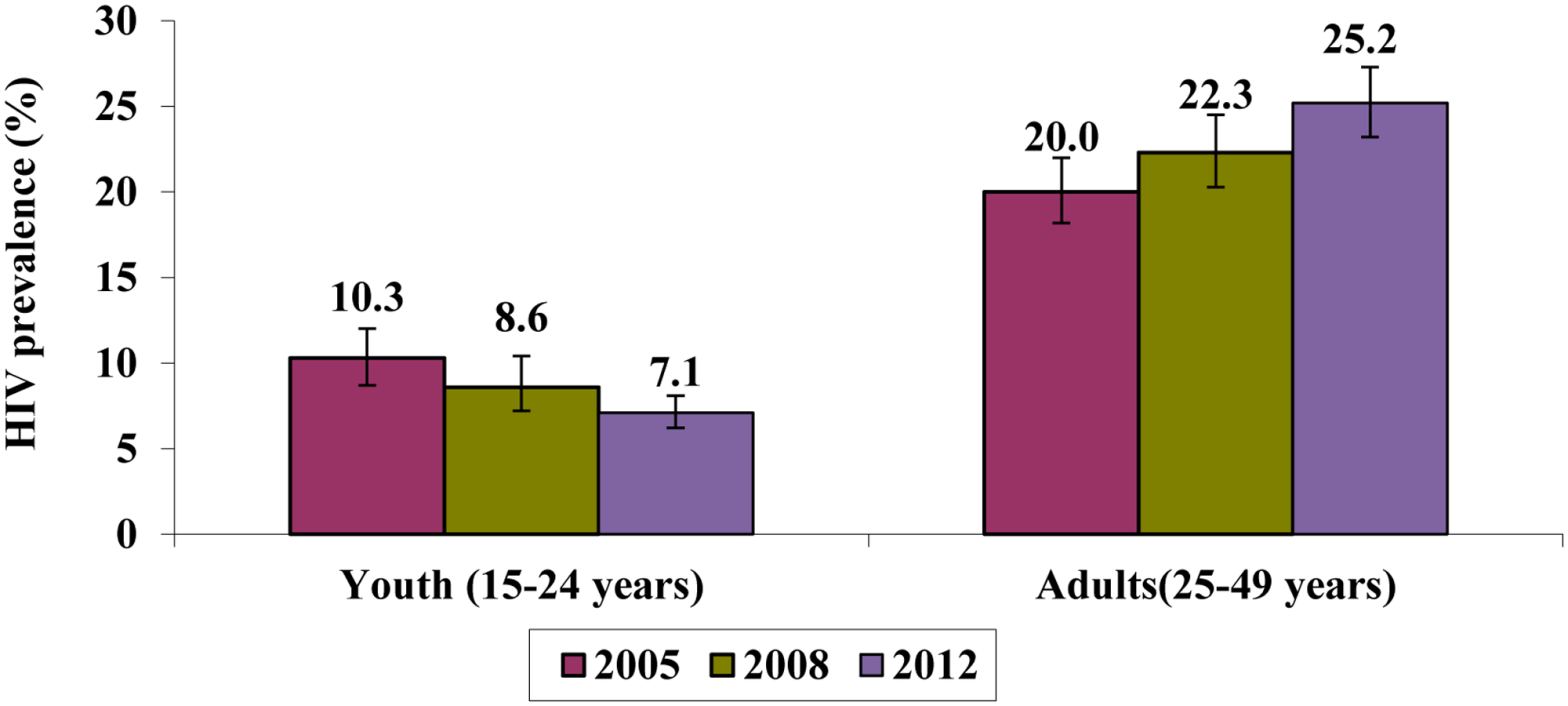
HIV prevalence trends by age group and survey year, South Africa 2005–2012. HIV prevalence among youth aged 15–24 years and adults aged 25–49 years estimated in the years 2005, 2008 and 2012. Source: Human Science Research Council Surveys [3].

Prevalence results from the 2005, 2008 and 2012 national HIV household surveys served as key input data to inform indirect model-based incidence estimation. Fig 2 shows the survey estimates of HIV prevalence trends by age group and survey year. There was a significant decline in HIV prevalence among persons aged 15–24 years from 10.3% (95% CI 8.7–12.0) in 2005 to 7.1% (95% CI 6.2–8.1) in 2012 (p<0.001). In contrast, among persons aged 25–49 years, prevalence increased significantly from 20.0% (95% CI 18.2–21.9) in 2005 to 25.2% (95% CI 23.2–27.3) in 2012 (p<0.001).

### Level of HIV incidence by estimation method, 2011/2012

**Fig 3:**
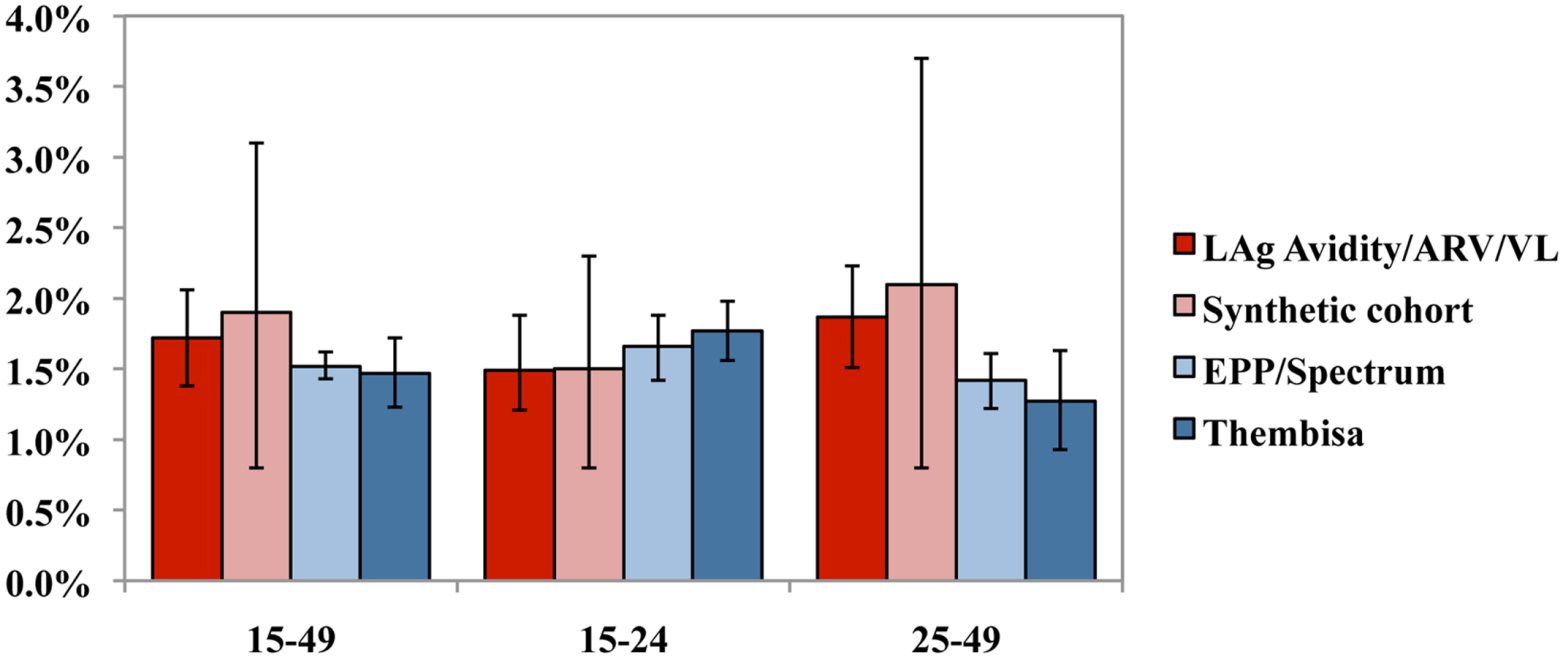
HIV incidence by age group and estimation method, South Africa 2012. 2012 HIV incidence rates (males and females combined) for the age groups 15–49 years, 15–24 years and 25–49 years provided by the four different estimation methods (LAg-Avidity/ARV/VL, Synthetic cohort, EPP/Spectrum, Thembisa). The error bars show the 95% uncertainty interval.

In Table 2, we compare national HIV incidence estimates by method. In addition, Fig 3 illustrates the main findings for 2012 by age group and estimation method. In 2012, we found no significant difference when we compared incidence generated by the assay-based approach, the synthetic cohort method, the Thembisa model and the EPP/Spectrum model. Assay-based HIV incidence was estimated at 1.72% (95% CI 1.38–2.06) for persons aged 15–49 years, but higher among females (2.28%; 95% CI 1.84–2.74) compared to males (1.21%; 95% CI 0.97–1.45). The synthetic cohort approach estimated HIV incidence to be 1.9% (95% CI 0.8–3.1) in the same age-group for the inter-survey period 2008–2012, with a less substantial difference between females (2.1%; 95% CI 1.0–3.4) and males (1.6%; 95% CI 0.6–2.7).

**Table 2:** Comparison of South African national HIV incidence estimates, 2005–2012.

Source	Method	Period	15–49 incidence % (95% CI)			15–24 incidence % (95% CI)			25–49 incidence % (95% CI)		
			Total	Males	Females	Total	Males	Females	Total	Males	Females
HSRC	LAg avidity/	2012	1.72	1.21	2.28	1.49	0.55	2.54	1.87	1.66	2.1
household	ARV testing/		(1.38–2.06)	(0.97–1.45)	(1.84–2.74)	(1.21–1.88)	(0.45–0.65)	(2.04–3.04)	(1.51–2.23)	(1.32–1.98)	(1.70–2.50)
survey	VL testing										
	Synthetic	2008–12	1.9	1.6	2.1	1.5	1.0	2.1	2.1	2.1	2.2
	cohort		(0.8–3.1)	(0.6–2.7)	(1.0–3.4)	(0.8–2.3)	(0.4–1.6)	(1.2–3.1)	(0.8–3.7)	(0.8–3.7)	(0.8–3.7)
		2005–08	1.9	1.6	2.2	2.3	1.4	3.5	1.5	1.8	1.1
			(0.8–3.3)	(0.6–3.0)	(1.0–3.6)	(1.2–3.5)	(0.5–2.3)	(2.1–4.9)	(0.4–3.0)	(0.6–3.5)	(0.1–2.5)
Thembisa	Mathematical	2011/12*	1.47	1.1	1.88	1.77	0.82	2.83	1.27	1.3	1.24
	model		(1.23–1.72)	(0.84–1.36)	(1.48–2.28)	(1.56–1.98)	(0.70–0.94)	(2.38–3.29)	(0.93–1.63)	(0.88–1.72)	(0.70–1.79)
		2008/09	1.79	1.43	2.19	1.96	0.95	3.1	1.67	1.78	1.57
			(1.49–2.09)	(1.11–1.75)	(1.70–2.67)	(1.70–2.23)	(0.81–1.09)	(2.52–3.68)	(1.25–2.10)	(1.25–2.31)	(0.91–2.22)
		2005/06	1.98	1.63	2.35	2.07	1.04	3.23	1.92	2.06	1.77
			(1.62–2.34)	(1.24–2.02)	(1.77–2.92)	(1.72–2.42)	(0.85–1.23)	(2.47–3.99)	(1.41–2.42)	(1.43–2.70)	(1.00–2.54)
EPP/Spectrum	Mathematical	2011/12*	1.52	1.29	1.78	1.66	0.91	2.5	1.42	1.52	1.31
(Version 5)	model		(1.43–1.62)	(1.21–1.37)	(1.67–1.90)	(1.42–1.88)	(0.69–1.10)	(2.23–2.76)	(1.22–1.61)	(1.16–1.84)	(1.17–1.45)
		2008/09	1.84	1.56	2.15	2.01	1.11	3.03	1.71	1.85	1.56
			(1.76–1.93)	(1.49–1.64)	(2.06–2.26)	(1.73–2.26)	(0.86–1.32)	(2.71–3.31)	(1.47–1.92)	(1.44–2.21)	(1.39–1.70)
		2005/06	2.01	1.70	2.35	2.19	1.21	3.32	1.86	2.03	1.69
			(1.92–2.10)	(1.62–1.78)	(2.24–2.46)	(1.89–2.44)	(0.93–1.44)	(2.99–3.59)	(1.60–2.07)	(1.57–2.42)	(1.52–1.83)

* Model HIV incidence estimates are for the period from mid-year to mid-year.

The mathematical models produced similar results to one another for the age group 15–49 years. The Thembisa model estimated incidence at 1.47% (95% CI 1.23–1.72), with HIV incidence significantly higher among females (1.88%; 95% CI 1.48–2.28) than males (1.10%; 95% CI 0.84–1.36), while the EPP/Spectrum model estimated incidence at 1.52% (95% CI 1.43–1.62), and, similar to Thembisa, produced incidence estimates that were higher among females (1.78%; 95% CI 1.67–1.90) than males (1.29%; 95% CI 1.21–1.37).

Among young persons aged 15–24 years, incidence was similar by estimation method, ranging from as low as 1.49% (95% CI 1.21–1.88) using the assay-based approach to as high as 1.77% (95% CI 1.56–1.98%) using the Thembisa model. Similar trends were observed by sex among persons aged 15–24 years, with female incidence ranging from as low as 2.1% (95% CI 1.2–3.1) using the synthetic cohort to as high as 2.83% (95% CI 2.38–3.29) using the Thembisa model, and significantly higher than incidence for males which ranged from a low of 0.55% (95% CI 0.45–0.65) using the assay-based approach to a high of 1.0% (95% CI 0.4–1.6) using the synthetic cohort approach. In contrast, among persons aged 25–49 years, incidence differed considerably by estimation method, with lower estimates generated by the Thembisa (1.27%; 95% CI 0.93–1.63) and EPP/Spectrum models (1.42%; 95% CI 1.22–1.61) compared with the assay-based (1.87%; 95% CI 1.51–2.23) and synthetic cohort methods (2.1%; 95% CI 0.8–3.7).

### Trends in HIV incidence by estimation method, 2005–2012

We observed different temporal trends in HIV incidence by estimation method between 2005 and 2012 for persons aged 15–49 years. Estimates generated by the synthetic cohort method indicate stable incidence: 1.9% (95% CI 0.8–3.3) between 2005 and 2008, and 1.9% (95% CI 0.8–3.1) between 2008 and 2012. Both the Thembisa and EPP/Spectrum models suggest a declining trend in HIV incidence between 2005 and 2012, with a percentage point (pts) change of -0.51% pts between 2005/2006 and 2011/2012 for the Thembisa model and -0.49% pts between 2005/2006 and 2011/2012 for the EPP/Spectrum model.

Among persons aged 15–24 years, a declining trend in HIV incidence was estimated by all three methods: the percentage point change was -0.80% pts between 2005–2008 and 2008–2012 for the synthetic cohort method; -0.30% pts between 2005/2006 and 2011/2012 for the Thembisa model; and -0.53% pts between 2005/2006 and 2011/2012 for the EPP/Spectrum model.

Among persons aged 25–49 years, we observed a similar pattern of declining incidence for the two mathematical models, with a percentage point change of −0.65% pts between 2005/2006 and 2011/2012 for the Thembisa model and -0.44% pts between 2005/2006 and 2011/2012 for the EPP/Spectrum model. This was not consistent with the synthetic cohort method, which produced a pattern suggestive of increasing incidence over time, with a percent change of +0.60% pts between 2005–2008 and 2008–2012.

### Performance of incidence testing algorithm by testing component

Table 3 compares the performance of the different components of the multi-assay recent infection testing algorithm we used for direct HIV incidence estimation. The 3-assay algorithm consisting of the LAg–Avidity assay in combination with antiretroviral drug testing and viral load testing estimated an incidence rate of 1.72% in the 15–49 years age group, with a zero false recent rate (FRR) assumed (see discussion for explanatory comments). When used in a single-assay format, the LAg–Avidity assay provided an incidence estimate of 3.58% for the same age group, which would require a FRR of 3.10% to be included in the incidence calculation in order to reproduce the incidence estimated by the 3-assay algorithm (LAg/ARV/VL). The LAg–Avidity assay in combination with antiretroviral drug testing (LAg/ARV) estimated an HIV incidence of 1.98% compared to 2.24% estimated by the Lag—Avidity assay in combination with viral load testing (LAg/VL). The computed false recent rates associated with these testing components, 0.40% for LAg/ARV and 0.86% for LAg/VL (assuming zero FRR for the 3-assay algorithm), are substantially smaller than the FRR of 3.1% found with the performance of the LAg–Avidity assay alone.

**Table 3:** Assay- based HIV incidence and FRR by testing component, South Africa 2012.

Algorithm	HIV incidence (15–49 age group)	FRR required to reproduce incidence estimate of full algorithm
LAg/ARV/VL	1.72%	-
LAg/ARV	1.98%	0.40%
LAg/VL	2.24%	0.86%
LAg only	3.58%	3.10%

Abbreviations: FRR: false recent rate; LAg: Limiting-Antigen Avidity assay (LAg-Avidity EIA); ARV: antiretroviral drug testing; VL: viral load testing

## Discussion

Given the evolving field of HIV incidence estimation and limitations of current methods for estimating incidence, our results suggest that a synthesis of multiple methods for estimating incidence in the same population is helpful in producing robust estimates of national HIV incidence levels and trends. More confidence can be placed in such results, as opposed to relying on findings from individual methods alone [26]. In the era of rapidly expanding antiretroviral treatment programs, HIV incidence estimation among different age groups is difficult but nevertheless critical for assessing the changing age-specific pattern of HIV prevalence.

Overall, the different incidence estimation methods were in remarkable agreement on 2012 HIV incidence estimates among persons aged 15–49 years. Though the direct assay-based method produced slightly higher estimates of incidence compared with mathematical models and slightly lower estimates of incidence compared to the synthetic cohort, the multi-method comparison shows similar levels and trends in HIV incidence, validating the results of the incidence testing algorithm and highlighting its utility in estimating incidence in cross-sectional settings.

There has been substantial progress over the past decade in the development and evaluation of HIV incidence assays [14, 27]. The LAg-Avidity EIA, used in an algorithm where assay-recent specimens are further tested for HIV RNA levels and for the presence of antiretroviral drugs, is currently the recommended approach to classify recent infections [28]. Our analysis of the performance of the different components of the incidence testing algorithm confirms the utility of testing for ART exposure and viral load to correct for the main sources of false recent misclassifications [29]. We have also demonstrated the relatively poor performance of the LAg-Avidity assay if applied in a single-assay format, requiring a false recent rate correction of 3.1% to reproduce the incidence estimate provided by the full algorithm. Out of 195 LAg-Avidity assay-recent specimens, 106 (54.4%) were re-classified as non-recent infections after testing for antiretroviral treatment exposure and viral load. This algorithm can be performed using dried blot spot (DBS) specimens, which is an important advantage in large population-based surveys [15]. The use of self-report of ART exposure in an incidence testing algorithm may be highly unreliable, as has been shown by several studies [30, 31, 32].

Based on the latest recalibration of the LAg-Avidity EIA in different HIV-1 subtypes, we applied a normalized optical density (ODn) cutoff of 1.5 and the corresponding mean duration of recent infection of 130 days for the incidence estimation [15]. Our assay-based HIV incidence estimates seem to confirm the validity of the selected parameters in the incidence calculation. A recently published assessment by the Consortium for the Evaluation and Performance of HIV incidence Assays (CEPHIA) proposed an alternative MDRI of 177 days and a false recent rate of 1.3% for the LAg-Avidity EIA (1.5 ODn cutoff) in subtype C specimens which excluded treated subjects and elite controllers [33]. However, applying those parameters in the incidence calculation based on our testing algorithm would have resulted in a change of the incidence estimate from 1.72% (95% CI 1.38–2.06) to 0.66% (95% CI 0.04–1.28) in the 15–49 year age group—an estimate that is not supported by any of the epidemiological/mathematical models. A recent revision by the CEPHIA group suggested a MDRI of 140 days and a FRR between 0% and 0.5% for an algorithm that assessed the LAg-Avidity EIA (1.5 ODn cutoff) in combination with a viral load threshold of ≥ 1000 copies/ml [34]. This parameter setup would have produced incidence rates more in agreement with our results, with estimates varying from 1.31% to 1.60%.

We did not include a false recent rate in our incidence calculation, relying on the correction of potential “false recent” results by means of additional testing for antiretroviral drugs and viral load in LAg-Avidity EIA recent specimens. Incidence testing algorithms of this type can reduce the false recent rate to almost zero, as has been demonstrated in samples from individuals in the United States [35]. Although the debate has focused so far on the potential false recent rate, we may also have to consider potential ‘false long-term’ misclassifications associated with this testing algorithm. HIV seroconverters who have been exposed to early treatment may be misclassified as chronically infected individuals on ART, e.g. recently infected pregnant women enrolled in prevention of mother-to-child-transmission (PMTCT) programs or persons on post-exposure prophylaxis (PEP) and pre-exposure prophylaxis (PrEP) regimens [36]. However, the extent of these potential misclassifications in the context of the 2012 national household survey was most likely extremely small. Finally, we should note that antibody-based screening assays are unable to detect acute HIV infections in the pre-seroconversion window. In the recently conducted national household survey in Swaziland, 13 (0.1%) of the 12 338 HIV antibody-negative specimens were identified as HIV RNA positive recent infections [37]. Based on these considerations discussed above we decided not to apply a correction factor to the multi-assay algorithm-derived 2012 HIV incidence estimates at this point in time.

Two mathematical models produced consistent estimates of HIV incidence level and trend, despite important differences in model assumptions and calibration procedures. While the EPP/Spectrum model allows a fair degree of flexibility in the estimation of HIV incidence trends, the Thembisa model estimates of HIV incidence are to some extent constrained by assumptions about trends in sexual behavior. The models also differ in their assumptions regarding ART rollout, with Thembisa assuming greater ART uptake and estimating a greater reduction in AIDS mortality due to ART. While the Thembisa model is calibrated to age-specific HIV prevalence data and recorded death data, the EPP/Spectrum model is calibrated to HIV prevalence data for the entire 15–49 age group. Since HIV prevalence data are not disaggregated by age groups in the model, EPP/Spectrum is not able to capture a scenario in which the incidence trend for 15–24 year olds is different from the trend observed in 25–49 year olds. A future step will be for the models to use age-specific data in their inference of epidemic trends.

All incidence estimation methods produced similar estimates for 15–24 year olds, and were consistent in their estimates of trends. In the population aged 25–49 years, however, the discrepancies between modelled estimates and the 2012 survey-based estimates are more evident. The Thembisa model may have overestimated treatment exposure among older adults aged 25–49 years and, as a result, overestimated the impact of ART on HIV incidence reduction in this age group. In the Thembisa model, 40.6% of HIV-positive adults aged 25–49 were estimated to be on ART in 2012 compared to 31.2% treatment exposure measured in the HIV-positive blood samples [3].

While important differences were observed in HIV incidence levels by sex, age, and time in our analysis, the wide confidence intervals around the incidence estimates suggest that these differences should be interpreted with caution. The synthetic cohort model appears to produce more uncertain estimates than other modelling approaches. This is because the method is unconstrained—i.e. the incidence pattern estimated from the HIV prevalence data does not have to be consistent with any theory or hypothesis about epidemic dynamics. The synthetic cohort approach is more sensitive to sampling errors or other aberrations in the data compared to the mathematical models, which are constrained to follow smooth changes over time or changes that are consistent with an underlying theory of epidemic dynamics. For example, if the previous survey in 2008 slightly underestimated adult HIV prevalence compared to the results obtained by the more representative 2012 survey design, the underestimate would particularly affect the synthetic cohort approach. The incidence estimate among persons aged 15–49 years produced by this method would be 1.5% instead of 1.9% if the bias in the 2008 prevalence estimate was about one percentage point overall and uniform by age and sex. Moreover, many assumptions required by this method are not readily identifiable from the input data, meaning that uncertainties, especially about ART, are propagated to the results.

Our results confirm that South Africa ranks first in the world in the annual number of new HIV infections [1]. The HIV incidence rates presented in this analysis suggest that about 1 000 new infections occurred each day among South Africans aged 15–49 years in 2012. Although the declining trend in HIV incidence between 2005 and 2012 observed among young adults aged 15–24 years is encouraging, the incidence rates still remain at unacceptably high levels, especially among female youth. The current National Strategic Plan on HIV, STIs and TB 2012–2016 states as its primary goal a reduction in new infections of at least 50% [38]. This would require a reduction in HIV incidence well below the 1% level among persons aged 15–49 years by 2016—a considerable challenge given the transmission dynamics that still prevail in the country. The 2012 survey findings indicated that there was a drop in condom use at last sex, an increase in the proportion of people reporting multiple sexual partners, and an increase in the proportion of young women reporting age-disparate relationships [3]. South Africa needs to balance treatment and prevention with a strong focus on the reduction of new HIV infections in the sexually active population.
